# Assessment of Intensive Care Unit Laboratory Values That Differ From Reference Ranges and Association With Patient Mortality and Length of Stay

**DOI:** 10.1001/jamanetworkopen.2018.4521

**Published:** 2018-11-09

**Authors:** Patrick D. Tyler, Hao Du, Mengling Feng, Ran Bai, Zenglin Xu, Gary L. Horowitz, David J. Stone, Leo Anthony Celi

**Affiliations:** 1 Department of Emergency Medicine, Beth Israel Deaconess Medical Center, Boston, Massachusetts; 2 Saw Swee Hock School of Public Health, National University Health System, National University of Singapore, Singapore; 3 School of Computer Science and Technology, University of Electronic Science and Technology of China, Chengdu, China; 4 Pathology and Laboratory Medicine, Tufts University School of Medicine, Boston, Massachusetts; 5 Departments of Anesthesiology and Neurosurgery, University of Virginia School of Medicine, Charlottesville; 6 Department of Pulmonary, Critical Care, and Sleep Medicine, Beth Israel Deaconess Medical Center, Boston, Massachusetts; 7 Division of Health Sciences and Technology, Harvard–Massachusetts Institute of Technology, Cambridge

## Abstract

**Importance:**

Laboratory data are frequently collected throughout the care of critically ill patients. Currently, these data are interpreted by comparison with values from healthy outpatient volunteers. Whether this is the most useful comparison has yet to be demonstrated.

**Objectives:**

To understand how the distribution of intensive care unit (ICU) laboratory values differs from the reference range, and how these distributions are related to patient outcomes.

**Design, Setting, and Participants:**

Cross-sectional study of a large critical care database, the Medical Information Mart for Intensive Care database, from January 1, 2001, to October 31, 2012. The database is collected from ICU data from a large tertiary medical center in Boston, Massachusetts. The data are collected from medical, cardiac, neurologic, and surgical ICUs. All patients in the database from all ICUs for 2001 to 2012 were included. Common laboratory measurements over the time window of interest were sampled. The analysis was conducted from March to June 2017.

**Main Outcomes and Measures:**

The overlapping coefficient and Cohen standardized mean difference between distributions were calculated, and kernel density estimate visualizations for the association between laboratory values and the probability of death or quartile of ICU length of stay were created.

**Results:**

Among 38 605 patients in the ICU (21 852 [56.6%] male; mean [SD] age, 74.5 [55.1] years), 8878 (23%) had the best outcome (ICU survival, shortest quartile length of stay) and 3090 (8%) had the worst outcome (ICU nonsurvival). Distribution curves based on ICU data differed significantly from the hospital standard range (mean [SD] overlapping coefficient, 0.51 [0.32-0.69]). All laboratory values for the best outcome group differed significantly from those in the worst outcome group. Both the best and worst outcome group curves revealed little overlap with and marked divergence from the reference range.

**Conclusions and Relevance:**

The standard reference ranges obtained from healthy volunteers differ from the analogous range generated from data from patients in intensive care. Laboratory data interpretation may benefit from greater consideration of clinically contextual and outcomes-related factors.

## Key Points

**Question:**

Should laboratory abnormalities in critically ill patients be interpreted using a reference interval generated from healthy outpatient controls?

**Findings:**

This cross-sectional study found associations between worst first-day values for common laboratory tests and mortality and length of stay for patients in the intensive care unit. Results showed substantial differences between the hospital reference interval and the reference intervals generated by data from patients in the intensive care unit.

**Meaning:**

Laboratory value ranges from critically ill patients deviate significantly from those of healthy controls; therefore, the strategy for interpretation of these laboratory values may benefit from a more contextual, probabilistic, and outcomes-based approach.

## Teaser

This cross-sectional study uses data from the Medical Information Mart for Intensive Care database to investigate how laboratory values of patients in the intensive care unit (ICU) differ from the standard reference range and to examine whether these differences are associated with mortality and length of stay in the ICU.

## Introduction

Critically ill patients have numerous laboratory abnormalities. The laboratory reference intervals that define normal values are established by sampling healthy outpatients; these intervals are also used to define abnormal values in patients in the intensive care unit (ICU).^1^ However, whether this is a valid or useful comparison has never been shown experimentally, and what values should count as abnormal in the critically ill population is unknown.

Our instinct and training as clinicians is to normalize abnormal values. However, there is no evidence that this approach is correct in all cases; at worst, it may even be harmful. There are instances in which correcting a laboratory abnormality is unhelpful or even causes increased mortality.^2,3,4,5,6^ Even seemingly appropriate responses, such as returning the glucose or hemoglobin to normal, can have unpredictable effects.^5,6^ Our understanding of the meaning of laboratory values in the ICU and how we should interpret and act on them is incomplete.

An editorial by Manrai et al^7^ addresses this question: “In the era of precision medicine,” they ask, “who is normal?” They note that “with the evolution of medicine into fully personalized or ‘precision’ medicine and the availability of large-scale data sets, there may be interest in trying to match each person to an increasingly granular normal reference population.” But what should we do with these new reference standards? The answer, the authors write, “would require studies that assess the outcomes of individuals with laboratory measurements classified as normal with one system vs abnormal with another. Outcomes could include both natural history and treatment benefits and harms.”

To study this further, we examined the laboratory values of critically ill patients over a 12-year period. We compared our hospital’s laboratory reference interval with one generated from data from patients in the ICU and performed data visualization that explored the association between laboratory values of interest and mortality. We hypothesized that the distributions of the laboratory values for critically ill patients would diverge from the hospital reference interval, and that there might be differences in the values among critically ill patients that could be associated with different outcomes.

## Methods

### Design

We conducted a single-center, retrospective, cross-sectional study with data visualization that focused on the first-day, most extreme (worst) measurement for laboratory values in a critically ill patient population. This study is reported in accordance with the Strengthening the Reporting of Observational Studies in Epidemiology (STROBE) reporting guideline.^8^ The study was approved by the institutional review boards of the Massachusetts Institute of Technology and Beth Israel Deaconess Medical Center and was granted a waiver of informed consent.

### Setting

We used the Medical Information Mart for Intensive Care (MIMIC) database, a collaboration between the Beth Israel Deaconess Medical Center and the Laboratory for Computational Physiology at the Massachusetts Institute of Technology. The database contains granular, deidentified ICU data from the Beth Israel Deaconess Medical Center. The data have been generated from more than 70 intensive care unit beds with medical, surgical, cardiac, and neurological patients. We used the latest data version, MIMIC-III (version 1.4), which contains deidentified data associated with 53 423 ICU admissions.^9^

### Participants

The analysis was conducted from March to June 2017. We selected all adult patients in MIMIC-III between January 1, 2001, and October 31, 2012 (N = 38 605). Patients were categorized into 5 groups based on ICU mortality and ICU length of stay (LOS). We stratified ICU survivors into quartiles based on their LOS; those in the first quartile, the shortest LOS, were defined as having the best outcome, and patients who died in the ICU were defined as having the worst outcome. Our analysis involved comparisons between the hospital reference interval and an interval generated by the ICU cohort; between the hospital interval and the best and worst outcome groups; and between the best and worst outcome groups. We extracted only data associated with each patient’s first ICU admission to ensure independence between data points.

### Primary Analysis

For each stay, we extracted worst first-day results for a panel of laboratory tests routinely ordered for patients in the ICU. We focused on clinically relevant laboratory values: minimum for albumin, ionized calcium, hemoglobin, and platelets; maximum for lactate; and both minimum and maximum for bicarbonate, blood urea nitrogen, creatinine, calcium, magnesium, phosphate, potassium, sodium, glucose, and white blood cell count. Missing data were handled as follows: if a patient had values for all the tests except albumin, the patient was included in all analyses except that for albumin. The proportion of patients missing each laboratory test is shown in the eAppendix in the Supplement; albumin was ordered for only 41% of patients, free (ionized) calcium for 50%, and serum lactate for 60%, with the remainder of the study laboratory tests ordered for more than 80% of patients.^10^

To evaluate the primary hypothesis (all distributions would differ significantly from the reference interval) and secondary hypothesis (distributions would be significantly different between best and worst outcome groups), probability distributions were generated for each laboratory value, stratified by cohort. For each graph of best or worst probability distributions, we calculated the degree of overlap and of divergence (see Statistical Analysis for details).

We then evaluated whether specific values for a given test corresponded to a patient’s probability of 1 of the 5 outcomes described. We calculated these probabilities and displayed them in heat maps as described in the Statistical Analysis section.

### Statistical Analysis

The data extraction algorithm was programmed and executed in PostgreSQL (PostgreSQL Global Development Group), and the statistical analytics were conducted in R (R Project for Statistical Computing) and Python. The queries were stored on a public repository through GitHub.^10^

The best and worst outcome populations were evaluated using descriptive statistics. In comparing baseline characteristics of these groups, continuous variables were analyzed using Kruskal-Wallis tests and categorical variables were analyzed using χ^2^ tests. Both tests were conducted with a 2-sided statistical significance level of .05.

Next, we compared the distribution of the laboratory test results of patients in the ICU against the corresponding hospital reference interval. For more effective comparisons, a kernel density estimation function was applied to smooth out the histogram diagrams. Kernel density estimation is a nonparametric method used to estimate the probability density functions of random variables.^11^ In the estimated probability density plot, the normal intervals for the laboratory test values were indicated by 2 vertical dotted lines. As the normal intervals for hospital reference purposes are defined as the central 95% interval among the normal cohort, the 95% intervals were calculated and compared for the groups of patients in the ICU. To reduce the effect of outlier values, the 95% intervals were calculated by excluding the extreme 2.5% from both ends of the distributions. We used the Wilcoxon signed rank test to test whether the differences between the mean values of our ICU population and of the reference intervals were statistically significant.

To quantify the divergence (or overlap) between the laboratory test result distributions from our ICU population and the reference interval, we calculated the overlapping coefficient (OVL) and the Cohen standardized mean difference (SMD).^12,13^ The OVL is intuitive—an OVL of 0 represents complete nonoverlap, and 1 represents complete overlap. The SMD is a measure of differences between 2 groups. It tells the size of the difference between the means relative to the variability observed in the 2 groups. Like the *z* score in standard normal distribution, the value of SMD stands for the difference in units of standard deviation. For example, an SMD of −2.03 on albumin minimal values between all ICU outcomes and the standard normal interval means that the average albumin minimal values of all patients in the ICU are 2.03 SDs less than the average of the standard normal interval. An SMD of 0 means that there was no difference between the means of 2 groups; less than 0.2 is a small effect size, 0.2 to 0.8 is a moderate effect size, and greater than 0.8 is a large effect size.

The relative proportions of the 5 outcome groups for specific test values were also calculated and graphically summarized into a stacked plot, or heat map. These stacked plots help visualize the relative likelihood for patients’ laboratory values to fall into 1 of the outcome groups. Extreme values of laboratory test results that account for less than 5% of the population were excluded, as there were too few data points in these regions to achieve a stable and robust estimation of the proportions.

## Results

We sampled the initial ICU admission data of 38 605 unique patients (21 852 [56.6%] male; mean [SD] age, 74.5 [55.1] years). The characteristics of the main study population, comparing patients with the best and worst outcomes, are displayed in Table 1. The best outcome group comprised 8878 patients (23%), and 3090 patients (8%) were in the worst group. Compared with the best group, the worst group was older, with a median (IQR) age of 73.2 (59.8-82.6) years vs 63.1 (49.9-73.8) years; sicker, with a median (IQR) Oxford Severity of Illness score of 41 (35-48) vs 27 (22-32); and had significantly higher rates for use of mechanical ventilation (87.2% vs 27.1%) and vasopressors (68.7% vs 16.2%).

**Table 1.** Baseline Characteristics of Main Study Population

Covariate	Median (IQR)		*P* Value
	Best Outcome (n = 8878)	Worst Outcome (n = 3090)	
Age at admission, y	63.05 (49.96-75.83)	73.18 (59.84-82.56)	<.001
Oxford Acute Severity of Illness score^a^	27 (22-32)	41 (35-48)	<.001
Female, No. (%)	3766 (42.4)	1465 (47.4)	<.001
Use of vasopressor, No. (%)	1437 (16.2)	2122 (68.7)	<.001
Use of mechanical ventilation, No. (%)	2404 (27.1)	2693 (87.2)	<.001
Service unit, No. (%)			
Cardiac intensive care unit	1278 (14.4)	489 (15.8)	<.001
Cardiac surgical recovery unit	1760 (19.8)	217 (7.0)	
Medical intensive care unit	3220 (36.2)	1359 (43.9)	
Surgical intensive care unit	1620 (29.5)	1025 (33.1)	
Laboratory test			
Albumin, g/dL			
Minimum	3.50 (3.00-4.00)	2.80 (2.30-3.40)	<.001
Bicarbonate, mEq/L			
Maximum	26.00 (24.00-28.00)	24.00 (2.00-27.00)	<.001
Minimum	24.00 (21.00-26.00)	2.00 (15.00-23.00)	<.001
Bilirubin, mg/dL			
Maximum	0.60 (0.40-1.00)	0.90 (0.50-2.20)	<.001
Creatinine, mg/dL			
Maximum	0.90 (0.80-1.20)	1.40 (0.90-2.30)	<.001
Glucose, mg/dL			
Maximum	156.00 (124.00-189.00)	177.50 (135.00-244.00)	<.001
Minimum	107.50 (94.00-130.00)	133.00 (102.00-179.00)	<.001
Hemoglobin, g/dL			
Minimum	10.70 (9.10-12.30)	9.90 (8.40-11.60)	<.001
Lactate, mmol/L			
Maximum	2.00 (1.40-2.90)	3.60 (2.00-7.20)	<.001
Magnesium, mEq/L			
Maximum	1.65 (1.56-1.89)	1.81 (1.65-2.06)	<.001
Minimum	1.56 (1.40-1.65)	1.48 (1.32-1.73)	<.001
Phosphate, mg/dL			
Maximum	3.60 (3.10-4.20)	4.30 (3.40-5.80)	<.001
Minimum	3.20 (2.70-3.80)	3.30 (2.50-4.40)	<.001
Platelet, ×10^3^/μL			
Minimum	200.00 (149.00-257.00)	172.50 (102.00-244.00)	<.001
Potassium, mEq/L			
Maximum	4.40 (4.10-5.00)	4.70 (4.20-5.40)	<.001
Minimum	3.80 (3.50-4.00)	3.70 (3.30-4.20)	<.001
Sodium, mEq/L			
Maximum	140.00 (138.00-142.00)	141.00 (138.00-144.00)	<.001
Minimum	137.00 (135.00-139.00)	137.00 (133.00-14.00)	<.001
White blood cell count, ×10^3^/μL			
Maximum	11.40 (8.50-14.90)	15.00 (1.60-2.60)	<.001
Minimum	8.70 (6.50-11.40)	1.80 (6.90-15.30)	<.001
Calcium, mg/dL			
Maximum	8.70 (8.30-9.10)	8.60 (8.00-9.10)	<.001
Minimum	8.40 (7.90-8.80)	7.90 (7.20-8.50)	<.001
Ionized calcium, mmol/L			
Minimum	1.08 (1.02-1.12)	1.03 (0.93-1.11)	<.001

Abbreviation: IQR, interquartile range.

SI conversion factors: To convert albumin and hemoglobin to g/L, multiply by 10.0; bicarbonate, potassium, and sodium to mmol/L, multiply by 1.0; bilirubin to μmol/L, multiply by 17.104; creatinine to μmol/L, multiply by 88.4; glucose to mmol/L, multiply by 0.0555; magnesium to mmol/L, multiply by 0.50; phosphate to mmol/L, multiply by 0.323; platelet count and white blood cell count to ×10^9^, multiply by 1.0; and calcium to mmol/L, multiply by 0.25.

^a^ The Oxford Acute Severity of Illness score is a machine learning, algorithm-driven adaptation of the Acute Physiology and Chronic Health Evaluation score. The score ranges from 0 to approximately 100, with higher scores representing a worse outcome.

**Figure 1.**
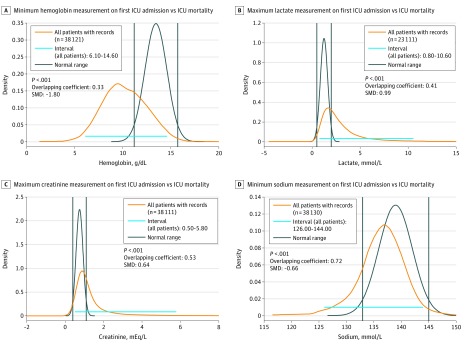
Comparison of Laboratory Values Between Intensive Care Unit (ICU) Cohort and the Reference Interval The distributions of our ICU cohort are shown in orange and the reference intervals are shown in black for minimum hemoglobin (A), maximum lactate (B), maximum creatinine (C), and minimum sodium (D). SMD indicates standardized mean difference.

A comparison of the distribution of values for several laboratory tests between the ICU cohort and the hospital reference interval is displayed in Figure 1. The intervals for all the laboratory tests were significantly different at the *P* < .001 level. Owing to space constraints, graphs are shown in Figure 1 only for the following laboratory tests: hemoglobin (minimum), lactate (maximum), creatinine (maximum), and sodium (minimum). Probability densities for the other tests are available in the eAppendix, eFigures 1 to 15, and the eTable in the Supplement.^10^

The probability distributions of laboratory test values between the best and worst outcomes were all significantly different from each other and from the hospital reference interval. Table 2 summarizes the overlapping and divergence coefficients of the hospital reference interval compared with the ICU cohort and best and worst outcome distributions. The mean (SD) OVL was 0.51 (0.32-0.69). As shown in the table, most of the laboratory tests have less than 0.8 overlap with the reference interval, with about half of them having less than 0.5 overlap. In addition, as shown from the SMDs between the entire ICU cohort and the best and worst outcome groups as compared with the hospital reference interval, most of the calculated effect sizes were greater than 0.8, indicating substantial disparity between the distributions.

**Table 2.** Overlap Between Laboratory Distributions of All Patients in the ICU, Best Outcome Patients, and Worst Outcome Patients With the Standard Reference Interval^a^

	All ICU Patients vs Standard Reference Interval		Best Outcome vs Standard Reference Interval		Worst Outcome vs Standard Reference Interval	
	OVL	SMD	OVL	SMD	OVL	SMD
Albumin						
Minimum	0.31	−2.03	0.40	−1.69	0.19	−2.57
Bicarbonate						
Maximum	0.72	−0.48	0.77	−0.41	0.54	−0.79
Minimum	0.46	−1.25	0.53	−1.09	0.33	−1.58
Bilirubin	0.50	0.90	0.57	0.76	0.28	1.28
Maximum						
Creatinine	0.53	0.64	0.57	0.51	0.33	1.04
Maximum						
Glucose						
Maximum	0.09	1.35	0.13	1.21	0.08	1.61
Minimum	0.40	0.94	0.42	0.96	0.29	0.93
Hemoglobin						
Minimum	0.33	−1.80	0.40	−1.54	0.30	−1.81
Lactate						
Maximum	0.41	0.99	0.47	0.95	0.23	1.28
Magnesium						
Maximum	0.85	0.14	0.86	−0.04	0.73	0.24
Minimum	0.61	−0.84	0.65	−0.81	0.59	−0.72
Phosphate						
Maximum	0.65	0.33	0.70	0.16	0.46	0.79
Minimum	0.57	−0.42	0.63	−0.43	0.49	0.04
Platelet count						
Minimum	0.53	−0.87	0.58	−0.75	0.48	−0.92
Potassium						
Maximum	0.72	0.72	0.77	0.61	0.65	0.82
Minimum	0.58	−0.99	0.62	−0.91	0.62	−0.71
Sodium						
Maximum	0.81	0.37	0.83	0.31	0.69	0.45
Minimum	0.72	−0.66	0.77	−0.59	0.68	−0.59
White blood cell count						
Maximum	0.34	0.88	0.41	0.78	0.26	0.69
Minimum	0.59	0.57	0.64	0.51	0.40	0.50
Calcium						
Maximum	0.52	−0.96	0.55	−1.02	0.49	−0.69
Minimum	0.33	−1.70	0.39	−1.56	0.27	−1.76
Ionized calcium						
Minimum	0.31	−1.83	0.27	−1.97	0.26	−1.66

Abbreviations: ICU, intensive care unit; OVL, overlapping coefficient; SMD, standardized mean difference.

^a^ Statistics showing overlap between the worst first-day laboratory values of the populations (all patients, best outcome, worst outcome) with the reference interval. The OVL is interpreted as follows: 0 represents complete nonoverlap and 1 represents complete overlap. The SMD stands for the difference in units of standard deviation. *P* values are omitted from the table because all OVL and SMD measurements were significant at a *P* threshold of <.05.

**Figure 2.**
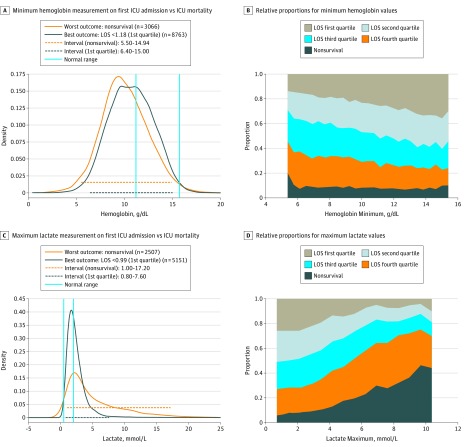
Results for the Minimum Hemoglobin and Maximum Lactate Measurements For panels A and C, the orange dashed line at the bottom of the graph represents the 95% probability distribution for patients with the worst outcome; the black dashed line represents the 95% probability distribution for those with the best outcome. For panels B and D, the stacked charts illustrate the relative proportions among all 5 outcome groups across a range of various laboratory values. ICU indicates intensive care unit; LOS, length of stay.

**Figure 3:**
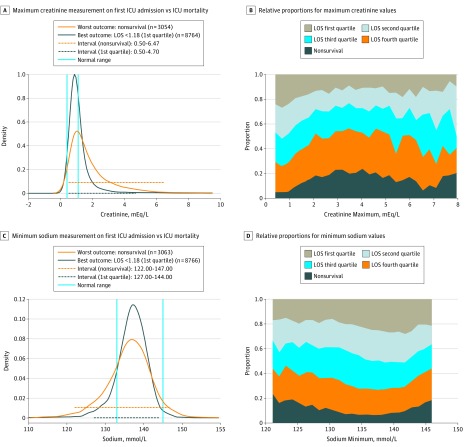
. Results for the Maximum Creatinine and Minimum Sodium Measurements For panels A and C, the orange dashed line at the bottom of the graph represents the 95% probability distribution for patients with the worst outcome; the black dashed line represents the 95% probability distribution for those with the best outcome. For panels B and D, the stacked charts illustrate the relative proportions among all 5 outcome groups across a range of various laboratory values. ICU indicates intensive care unit; LOS, length of stay.

Panels A and C in Figure 2 and Figure 3 display the distribution of values for the 2 outcome groups for several laboratory tests. Blue lines show the hospital reference interval. Beneath each probability distribution, 2 analogously color-coded horizontal lines demonstrate the central 95% interval for the 2 patient outcome cohorts. Taking the maximum serum lactate level as an example, the reference interval was 0.5 to 2.0 mmol/L. However, we observed that for this test, most patients from both outcome groups fell outside of the hospital reference interval. We also observed that once the maximum serum lactate level of the patient increased above 4.0 mmol/L, the chance of the worst outcome greatly surpassed that of the best.

We further investigated how the proportion (likelihood) of patients in the 5 predefined outcome groups changes across the range of laboratory values. The associated stacked charts for each laboratory test are shown in panels B and D of Figure 2 and Figure 3. Again, taking maximum serum lactate level as an example, we observed that the proportion of nonsurvival, indicated by the height of the dark gray zone, increased remarkably once the serum lactate value exceeded 4.0 mmol/L.

## Discussion

In this study, we found a significant difference between a reference interval based on ICU data compared with the hospital reference interval. This discrepancy was anticipated, as we compared worst first-day values from patients in the ICU with the values of healthy controls. This supports our hypothesis that what constitutes common, therefore normal, values in patients in the ICU is not congruent with what we call *normal* based on a very different population. In addition, we found significant divergence and lack of overlap between the distribution of values for patients with the best and worst outcomes, as well as between these 2 groups and the hospital reference interval. We also observed that specific values for a given laboratory test could be associated with the probability of a specified outcome (in this case, mortality and LOS). This more precise approach presents an alternative to the current practice of interpreting ICU laboratory tests based on comparison with the values of healthy controls.

As many authors have explored, the concept of what represents normal is a moving target.^14,15,16^ Additionally, the meaning of an abnormal test result depends to some extent on what information was being asked.^15,16^ In the intensive care unit, we use an approach to interpretation similar to what is done in the clinics and in the hospital: the results are compared with a reference interval that represents the central 95% of a healthy population.^15,16^ This approach sometimes leads to an intervention to correct the abnormality. There is an inherent assumption that the value is pathologically abnormal, when in fact it may represent a harmless epiphenomenon or, worse, a compensatory process.^17^

What we propose is a refinement of ideas that have been explored in depth by prior authors but that may only now be feasible as a result of very large data sets that include relevant clinical outcomes. As Manrai and colleagues^7^ write, “with the proliferation of large data sets emblematic of precision medicine, it is becoming feasible to study stratified variation and clinical outcomes at scale. Sample size limitations are no longer a challenge. However, the task of defining a ‘normal’ population becomes even more challenging. Who should define normality and using which criteria?” One could view our approach as epidemiological, in a sense, suggesting decision limits (similar to those of cholesterol) above which treatment is warranted to prevent adverse outcomes, and below which no action is necessary.^15^ Or the approach could be viewed as one based on refinements of reference intervals, which “are a biological characteristic of the population and, if defined with the necessary attention to the choice of subjects, with partitioning for sex, age, and other characteristics, combined with the use of standardized analytical methods producing results traceable to the reference measurement system, they could (and should) remain stable.”^15^ By using data from ICU populations with high-resolution features and various outcomes, including both long term (eg, survival) and intermediary (eg, occurrence of arrhythmia), we can more accurately reference a patient against an outcome of interest, rather than with respect to a healthy population. This enhancement will, with further research as described in the future directions that follow, help us better understand how different laboratory values should be interpreted. Using a fixed reference interval for critically ill patients may not the be the most effective strategy; rather, probabilistic interpretation of laboratory values may be more valuable in guiding treatment decisions and prognostication.

While our simple model represents only a static snapshot of a dynamic situation, this effort represents the first attempt to generate a personalized, data-driven strategy for laboratory value interpretation based on clinical context and outcomes. An accurate reference interval for patients in the ICU appears to differ substantially from the conventional reference interval, and the concept of one reference interval for all might need to be redefined. The central 95% distributions for the best and worst outcome groups are almost completely juxtaposed for some laboratory tests (Figure 2 and Figure 3). Data from patients in the ICU from the outcome categories may actually look more like each other than the data of healthy volunteers. Outside the central 95%, the different outcome groups generally diverge, with 1 or both tails of their graphs tending to differ more sharply.

We also wanted to better understand how a laboratory test result might translate into the patient’s probability of experiencing a particular outcome. We generated color-coded stack heat maps that display a visual summary of this concept. For each value, the probability of the best to worst outcome can be easily ascertained (panels B and D of Figure 2 and Figure 3). We do not maintain that a single individual laboratory value is producing these outcomes. However, such outcome-stratified displays may be a more informative and intuitive way for electronic health records to present data for clinical decision making.^18^

The most challenging questions relate to which values require intervention (as well as when and how). In some cases, such as patients with an extremely low hemoglobin level from a massive hemorrhage, intervention would seem to be essential because severe anemia directly results in organ dysfunction. However, the role for other interventions (for example, repleting electrolytes) is less clear; an abnormality may represent a compensatory process and, with repletion, we may be disrupting an adaptive response. For example, in a murine model of sepsis, removing lactate from the blood resulted in higher mortality; the authors posit that this may be because lactate is being used as a vital energy source.^19^ In other words, there has not been a practical way of determining which laboratory abnormalities in critically ill patients are clinically most relevant, and which, if actively corrected, will improve patient outcomes.^20^ In addition, as the well-studied intervention of red cell transfusion demonstrates, what makes physiologic sense in terms of optimizing oxygen delivery may not necessarily translate to improved recovery from critical illness.^6^

We understand that the impact of the different ranges on outcomes might be small in absolute terms but may still be significant in terms of affecting clinical outcomes, as in the case of adjusting target hemoglobin levels for transfusion. In addition, range modifications such as those we propose would predictably have a larger impact on clinical processes, as clinicians may not have to spend as much time detecting anomalous values and then attempting to correct these values into inappropriately applicable ranges for their patients. We realize that modifications of ranges based on our findings would add complexity to the process of establishing and calibrating normal ranges, but in an age that is increasingly moving toward the use of big data and precision medicine, it makes sense to use the data now available to increase the precision of what we do clinically.

Time, costs, and resources spent in unnecessary corrective processes would also be reduced. We suspect that reduced laboratory testing would also be a consequence as there would be less need to repeat anomalous values to establish their validity and less need to retest after therapeutic intervention. Furthermore, any iatrogenic intervention (eg, potassium replacement) has its risks of therapeutic misadventure, and associated adverse events might be reduced.

### Limitations

This work is exploratory, and thus has many limitations. First, it is a single-center study from 1 ICU database. Second, the approach described does not immediately lend itself to direct clinical translation. Crude outcomes such as length of stay and mortality are highly dependent on multiple interacting factors, including the patient’s specific pathology (or pathologies) and comorbidities, as well as interactions between laboratory values. However, our goal is not direct clinical translation, but rather to point out that there is limited overlap between our normal reference ranges and the laboratory values of patients, and that other approaches may be possible.

Third, our approach does not investigate the interactions with these other factors. It does not take into account specific pathologies or underlying comorbidities—steps that would certainly be necessary for this approach to be translated clinically.

Fourth, generating population-specific, outcome-based probability distributions for laboratory value interpretation requires the input of large amounts of data, as well as complex computational analysis. This represents a major barrier to clinical translation. Again, the purpose of our work is to highlight a potentially different mechanism to interpret laboratory values; the best way to integrate such an approach clinically remains to be seen.

Finally, 1 significant downside to using an approach such as this is that it represents a major departure from how clinicians are trained to interpret laboratory values—that is, to ask, “Is this value abnormal (high or low) and, if so, what does this mean?” The interpretation of values generated in our approach is not clear; thus, how to interpret these novel distributions and how to apply them in clinical decision making remains to be studied.

### Future Directions

This approach is intended to question what is normal when interpreting laboratory (or other) tests in a specific patient population. A probabilistic approach to interpreting laboratory results may be applied to other uses of a diagnostic test apart from prognostication, such as translation to the likelihood of a specific clinical event, complication, or disease being present. This approach might lend itself to various use cases, including estimation of the likelihood of life-threatening arrhythmia based on commonly monitored and oft-repleted serum electrolytes (eg, potassium and magnesium); the likelihood of *Clostridium difficile* infection based on the degree of white blood cell count elevation; or the likelihood of response to chemotherapy based on serum lactate dehydrogenase levels. Future research will focus on probabilistic interpretation of discrete outcomes and on probabilistic interpretation of dynamic trends of laboratory values and other clinical parameters.

## Conclusions

The use of a standard hospital laboratory value reference interval for data from patients in the ICU may be problematic in several respects. With probability density curves as graphical displays and a statistical approach appropriate for the comparison of these graphical results, we found that the probability distribution generated by data from patients in the ICU differed significantly from the standard reference interval. After stratifying patients in the ICU into 5 outcome categories based on mortality and LOS, we observed that reference intervals constructed from the 2 extreme (best and worst) outcome groups were significantly different from each other but still resembled each other more than they did the standard range. These findings suggest that a clinically contextual, more precise approach to the analysis of data from a defined clinical population may be more relevant and applicable to the interpretation of laboratory tests. While the added complexity of this approach would be overwhelming with paper medical record keeping, the digitization of medical data provides the opportunity to perform more precise analyses that have the potential to better inform decisions.

This study represents a conceptual approach toward using a more personalized and outcome-based interpretation of data in a designated patient population. We believe that the introduction of such an approach to clinical care and electronic health record design will prove to be beneficial, as the clinicians who manage data entry of medical records and who are accountable for patient outcomes must analyze, and make decisions based on, increasing amounts and types of clinical data.

## Notes

Supplement. **eAppendix.** Supplementary Missing Value Analysis **eFigure 1.** Summary of the Percentages of Missing Values for Various Lab Test Results **eFigure 2.** Albumin and Bicarbonate Minimum Result Plots **eFigure 3.** BUN and Calcium Minimum Result Plots **eFigure 4.** Chloride and Creatinine Minimum **eFigure 5.** Free Calcium and Lactate Minimum **eFigure 6.** Magnesium and Phosphate Minimum **eFigure 7.** Platelet and Potassium Minimum **eFigure 8.** WBC Minimum **eFigure 9.** Albumin and Bicarbonate Maximum **eFigure 10.** BUN and Calcium Maximum **eFigure 11.** Chloride and Free Calcium Maximum **eFigure 12.** Magnesium and Phosphate Maximum **eFigure 13.** Platelet and Potassium Maximum **eFigure 14.** Sodium and WBC Maximum **eFigure 15.** Hemoglobin Maximum **eTable.** Overlap Between Laboratory Distributions of All ICU Patients, Best Outcome Patients, and Worst Outcome Patients With the Standard Reference Interval
