# Supplementary material for: Assessment of Intensive Care Unit Laboratory Values That Differ From Reference Ranges and Association With Patient Mortality and Length of Stay
Source: JAMA Netw Open. 2018 Nov 9;1(7):e184521. doi: 10.1001/jamanetworkopen.2018.4521 (PMC6324400; doi:10.1001/jamanetworkopen.2018.4521)
Supplement: Supplement. — eAppendix. Supplementary Missing Value Analysis eFigure 1. Summary of the Percentages of Missing Values for Various Lab Test Results eFigure 2. Albumin and Bicarbonate Minimum Result Plots eFigure 3. BUN and Calcium Minimum Result Plots eFigure 4. Chloride and Creatinine Minimum eFigure 5. Free Calcium and Lactate Minimum eFigure 6. Magnesium and Phosphate Minimum eFigure 7. Platelet and Potassium Minimum eFigure 8. WBC Minimum eFigure 9. Albumin and Bicarbonate Maximum eFigure 10. BUN and Calcium Maximum eFigure 11. Chloride and Free Calcium Maximum eFigure 12. Magnesium and Phosphate Maximum eFigure 13. Platelet and Potassium Maximum eFigure 14. Sodium and WBC Maximum eFigure 15. Hemoglobin Maximum eTable. Overlap Between Laboratory Distributions of All ICU Patients, Best Outcome Patients, and Worst Outcome Patients With the Standard Reference Interval [file jamanetwopen-1-e184521-s001.pdf]

Supplementary Online Content

Tyler PD, Du H, Feng M, et al. Assessment of intensive care unit laboratory values that differ from reference ranges and association with patient mortality and length of stay. *JAMA Netw Open*. 2018;1(7):e184521. doi:10.1001/jamanetworkopen.2018.4521

eAppendix. Supplementary Missing Value Analysis

eFigure 1. Summary of the Percentages of Missing Values for Various Lab Test Results

eFigure 2. Albumin and Bicarbonate Minimum Result Plots

eFigure 3. BUN and Calcium Minimum Result Plots

eFigure 4. Chloride and Creatinine Minimum

eFigure 5. Free Calcium and Lactate Minimum

eFigure 6. Magnesium and Phosphate Minimum

eFigure 7. Platelet and Potassium Minimum

eFigure 8. WBC Minimum

eFigure 9. Albumin and Bicarbonate Maximum

eFigure 10. BUN and Calcium Maximum

eFigure 11. Chloride and Free Calcium Maximum

eFigure 12. Magnesium and Phosphate Maximum

eFigure 13. Platelet and Potassium Maximum

eFigure 14. Sodium and WBC Maximum

eFigure 15. Hemoglobin Maximum

eTable. Overlap Between Laboratory Distributions of All ICU Patients, Best Outcome Patients, and Worst Outcome Patients With the Standard Reference Interval

This supplementary material has been provided by the authors to give readers additional information about their work.

© 2018 Tyler PD et al. *JAMA Network Open.*

Supplementary Online Content

eAppendix. Supplementary Missing Value Analysis

For each ICU stay, we extracted worst first day results for a panel of laboratory tests routinely ordered for ICU patients. Missing data in laboratory tests was handled as follows: if a patient had values for all the tests except albumin, the patient was included in all analyses except that for albumin. The proportion of patients missing each lab test are shown in eFigure 1. Albumin was ordered for only 41% of patients; free (ionized) calcium for 50%; serum lactate for 60%; with the remainder of the study laboratory tests ordered for more than 80% of patients.

eFigure 1: Summary of the percentages of missing values for various lab test results.

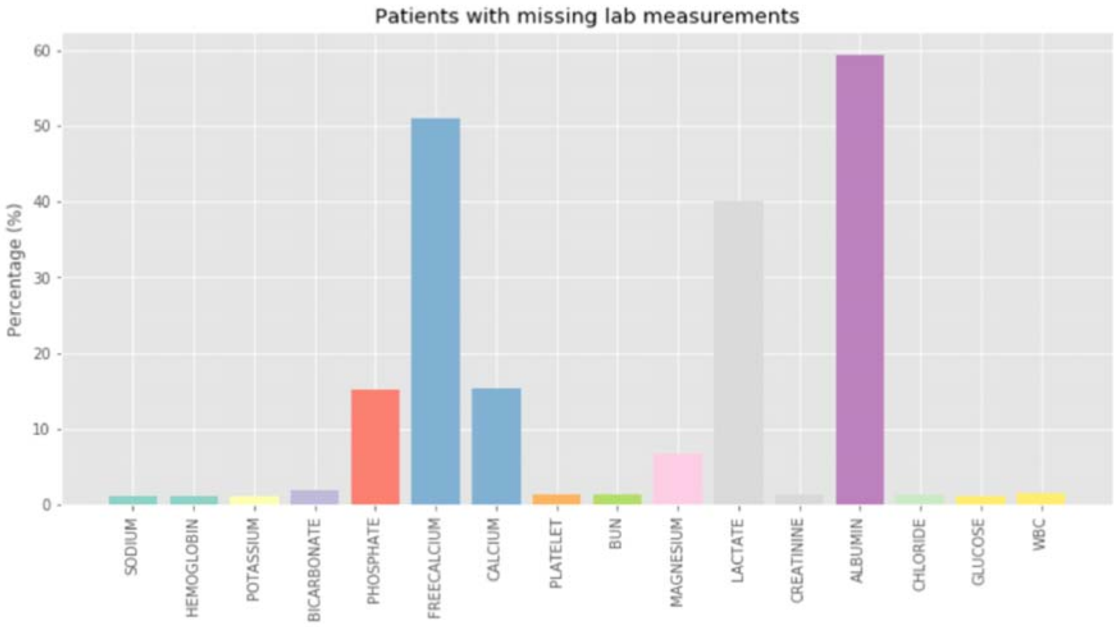

Supplementary Results

For each stay, we extracted worst first day results for a panel of laboratory tests routinely ordered for ICU patients. We focused on clinically relevant laboratory values: minimum for albumin, ionized calcium, hemoglobin, and platelets; maximum for creatinine and lactate; and both minimum and maximum for bicarbonate, calcium, magnesium, phosphate, potassium, sodium, and white blood cell count. The laboratory tests included in manuscript are excluded in the supplementary results.

Minimum Values

eFigure2: Albumin and Bicarbonate minimum result plots

© 2018 Tyler PD et al. *JAMA Network Open.*

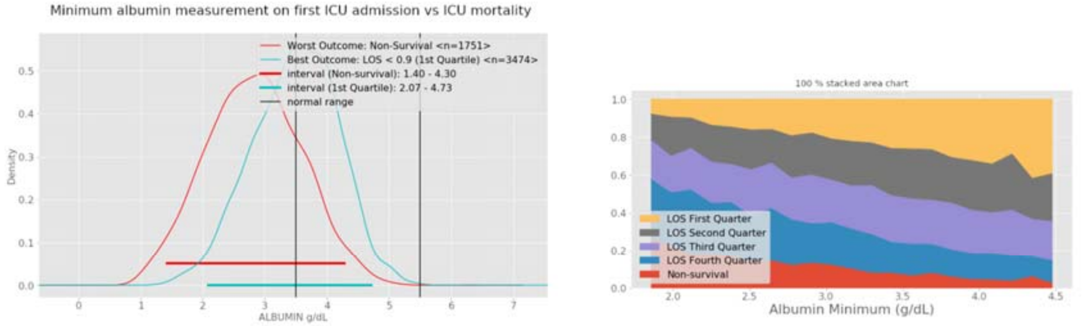

Albumin Minimum (g/dL)

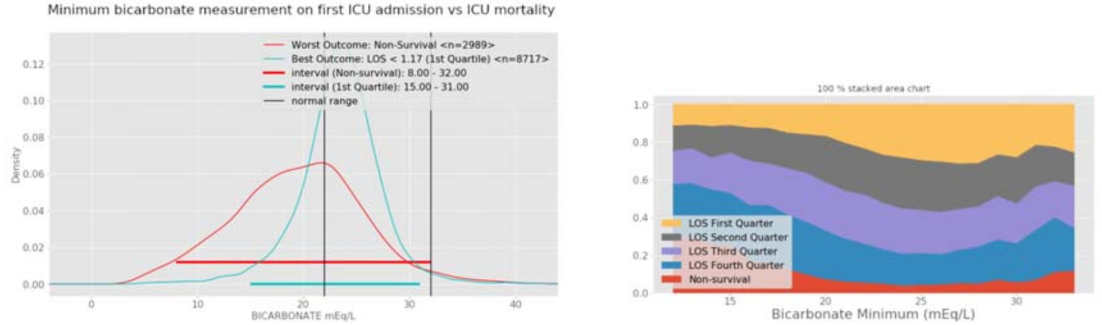

Bicarbonate Minimum (mmol/L)

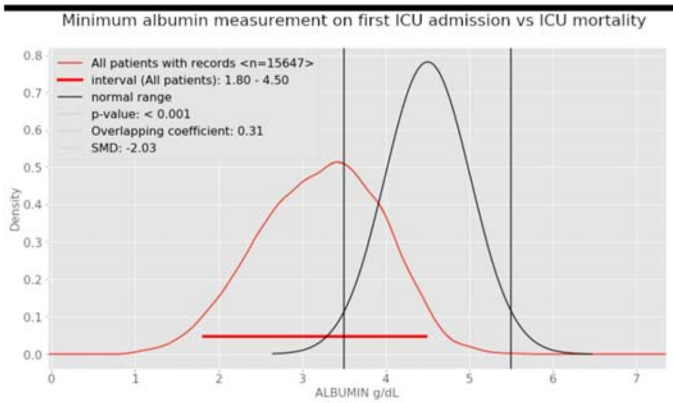

Albumin Minimum (g/dL)

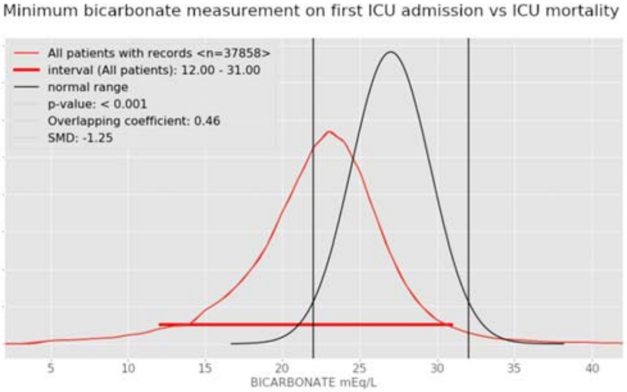

Bicarbonate Minimum (mEq/L)

© 2018 Tyler PD et al. *JAMA Network Open.*

eFigure 3: BUN and Calcium minimum result plots

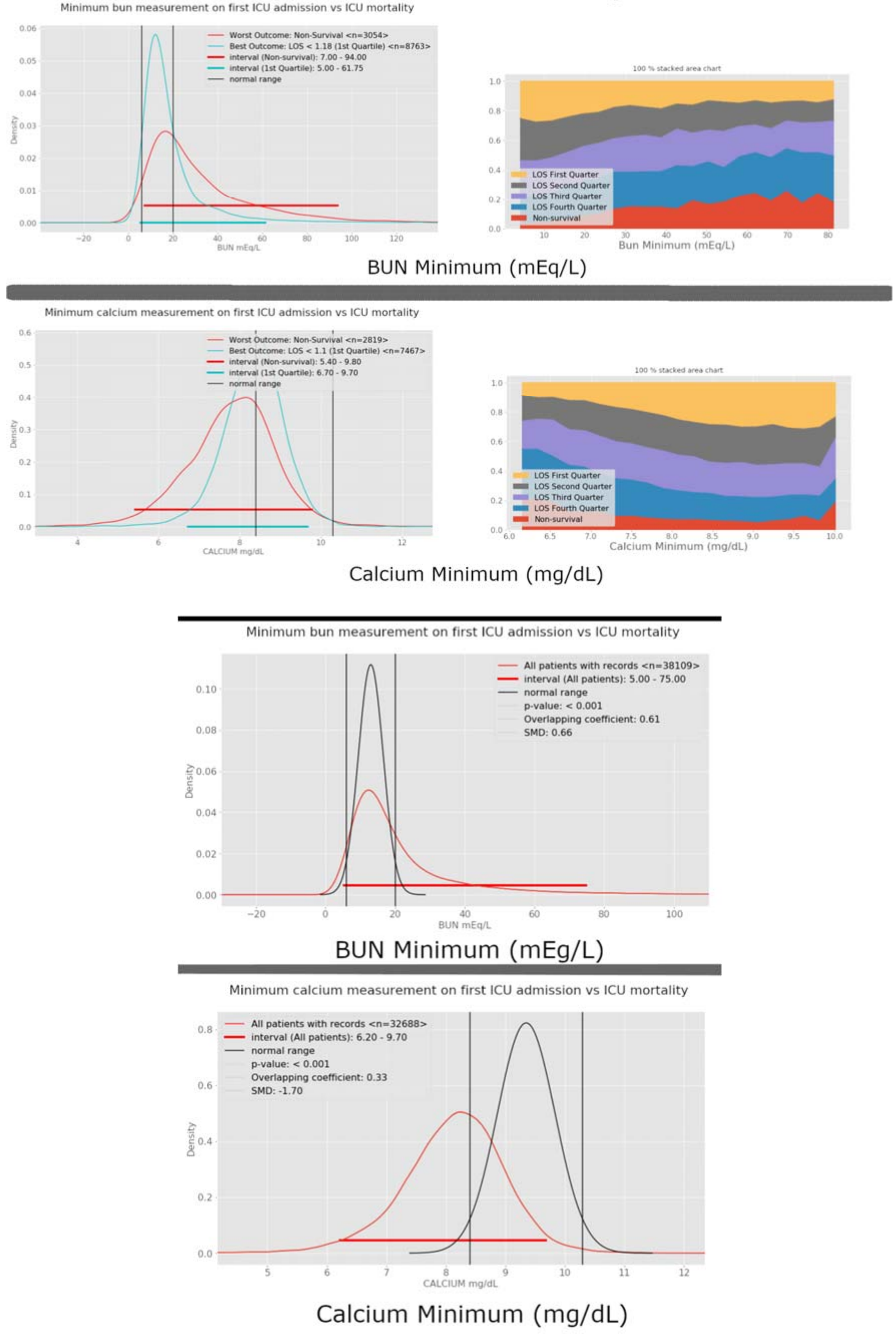

© 2018 Tyler PD et al. *JAMA Network Open.*

eFigure 4: Chloride and Creatinine minimum

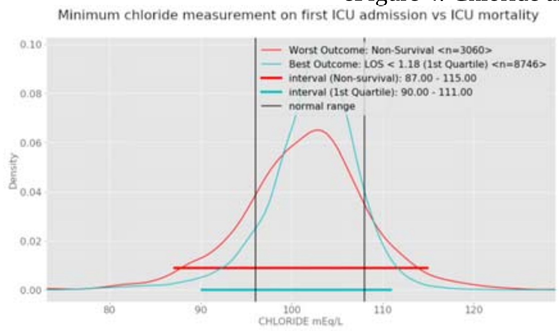

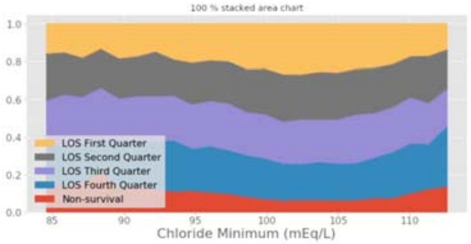

Chloride Minimum (mEq/L)

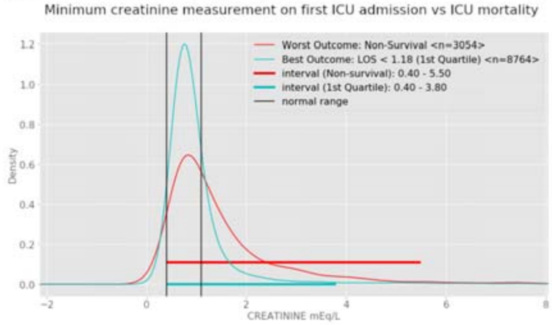

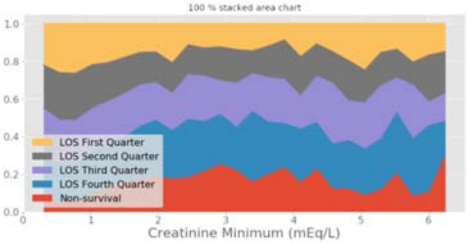

Creatinine Minimum (mEq/L)

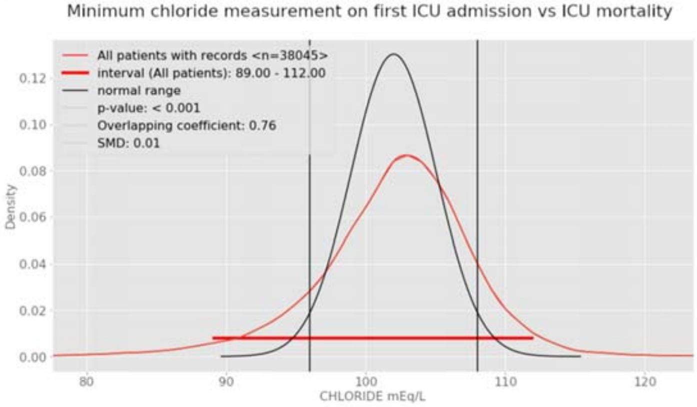

Chloride Minimum (mEq/L)

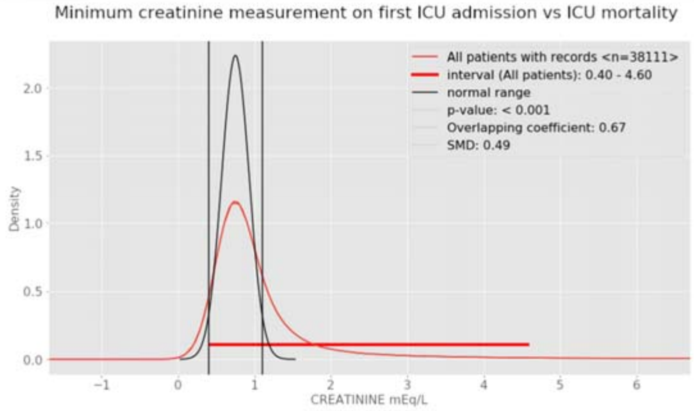

Creatinine Minimum (mEq/L)

© 2018 Tyler PD et al. *JAMA Network Open.*

eFigure 5: Free calcium and lactate minimum

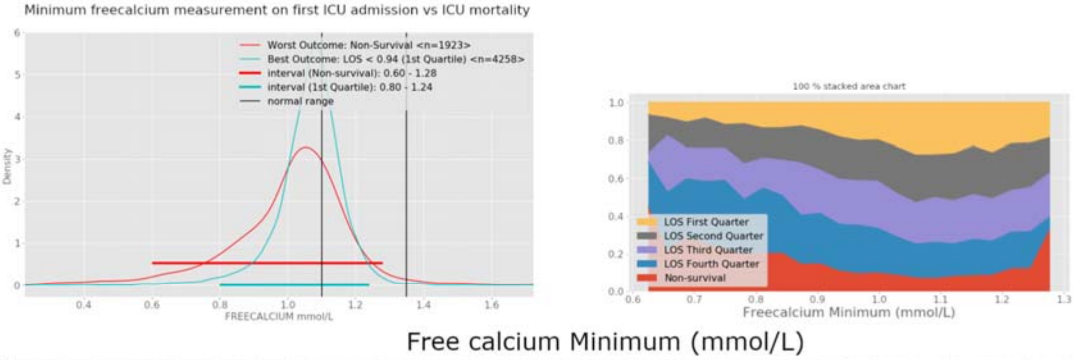

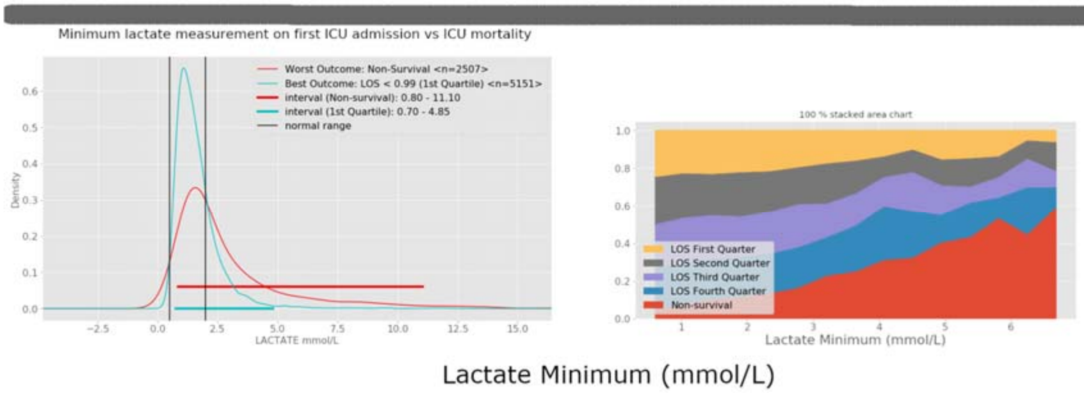

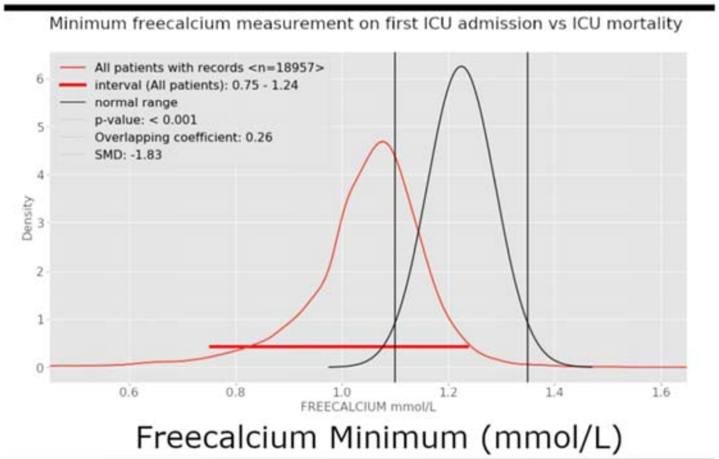

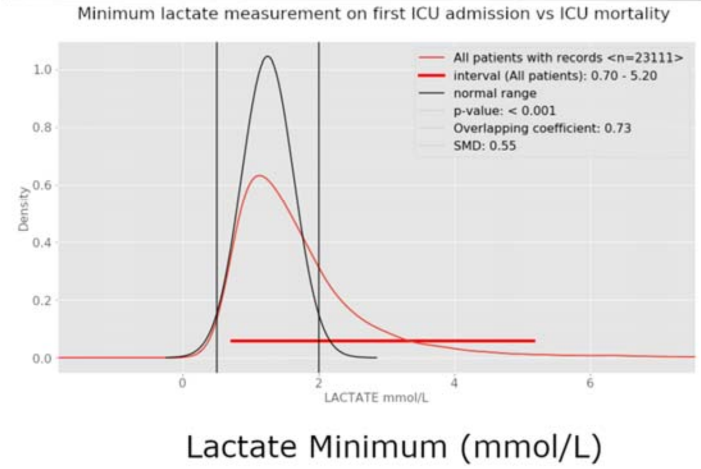

© 2018 Tyler PD et al. *JAMA Network Open.*

eFigure 6: Magnesium and Phosphate minimum

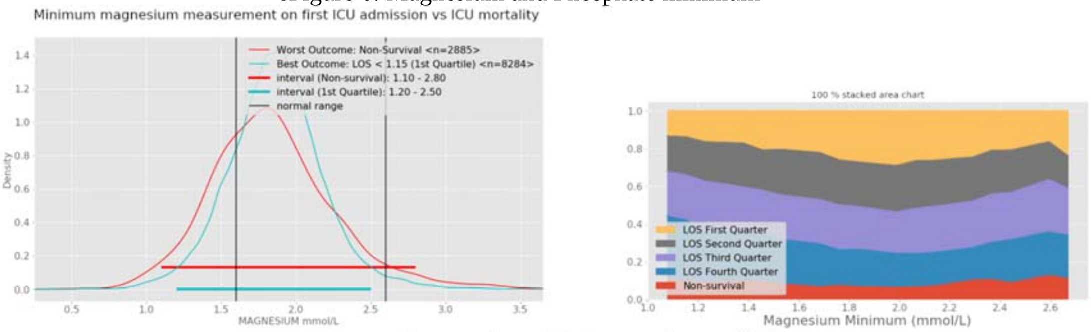

Magnesium Minimum (mmol/L)

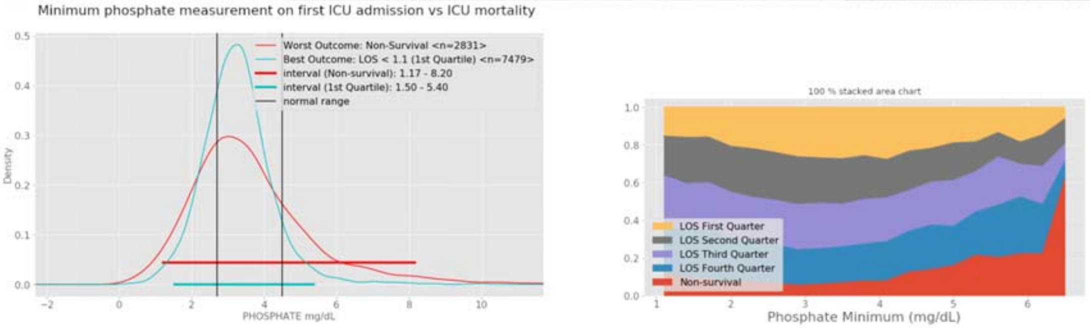

Phosphate Minimum (mg/dL)

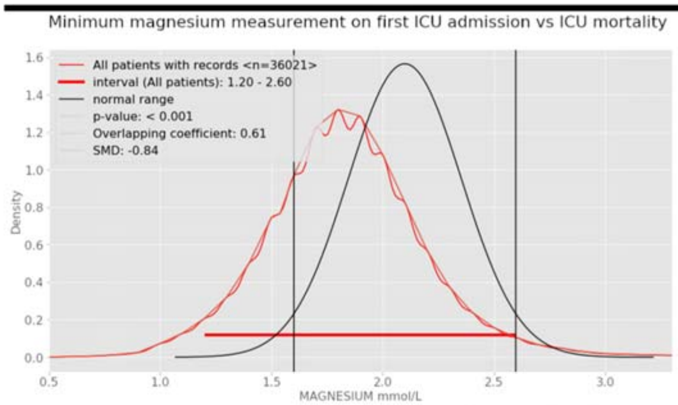

Magnesium Minimum (mmol/L)

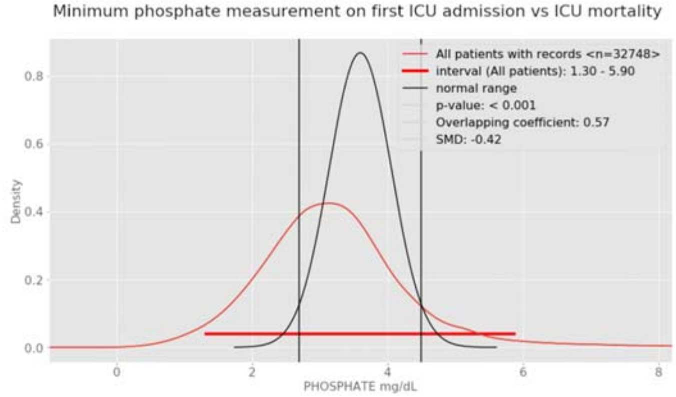

Phosphate Minimum (mg/dL)

© 2018 Tyler PD et al. *JAMA Network Open.*

eFigure 7: Platelet and Potassium minimum

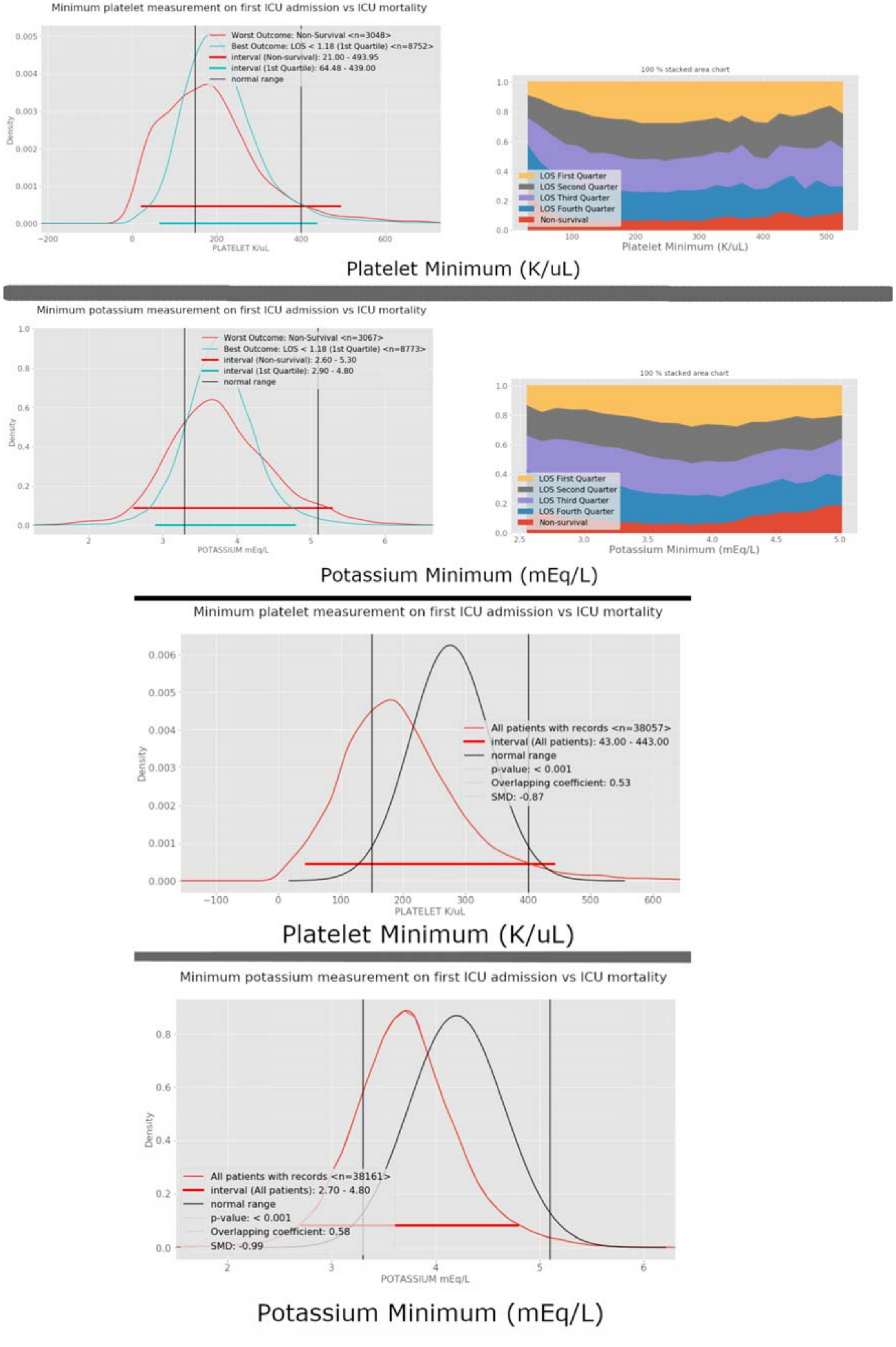

© 2018 Tyler PD et al. *JAMA Network Open.*

eFigure 8: WBC minimum

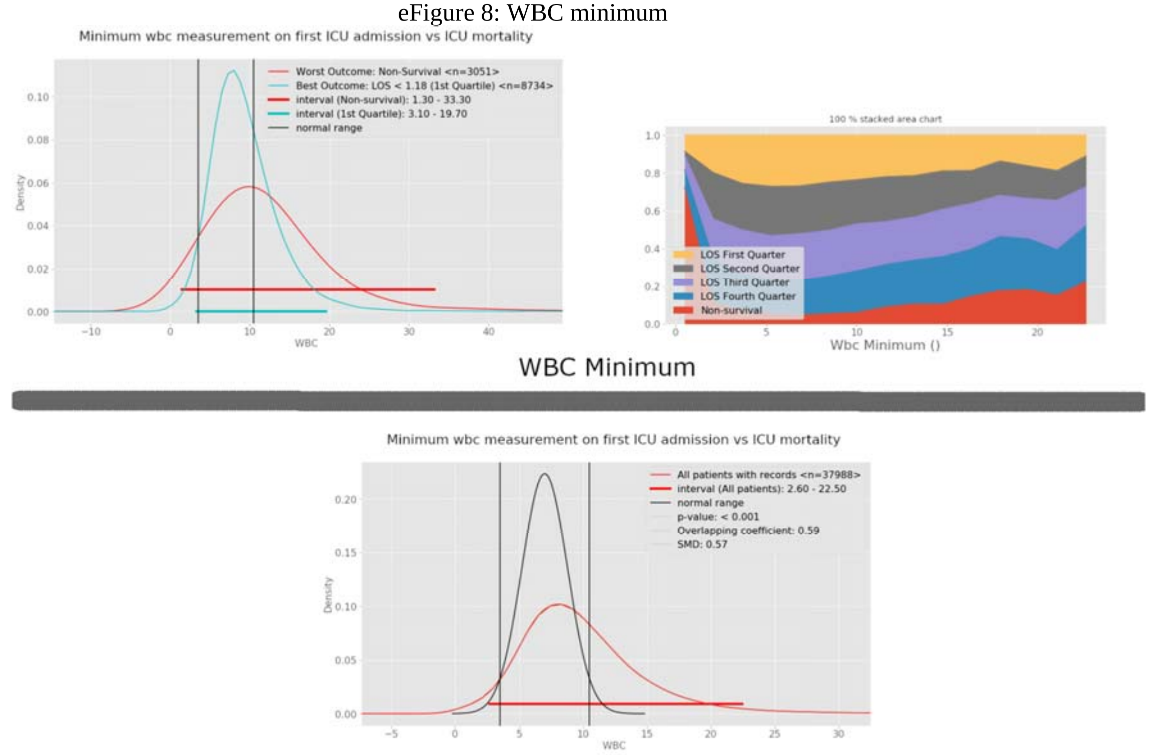

© 2018 Tyler PD et al. *JAMA Network Open.*

Maximum Values

eFigure 9: Albumin and Bicarbonate maximum

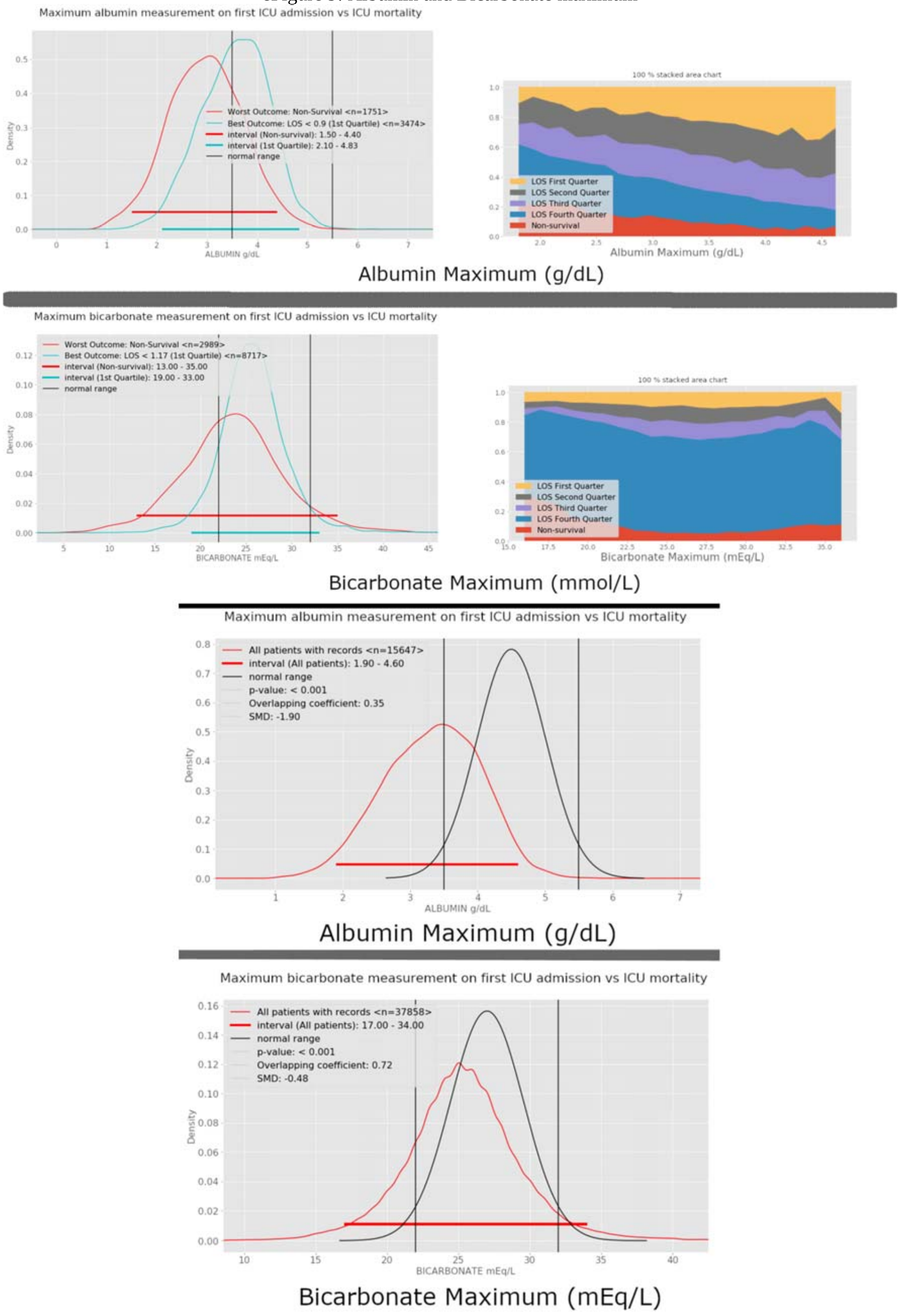

© 2018 Tyler PD et al. *JAMA Network Open.*

eFigure 10: BUN and Calcium maximum

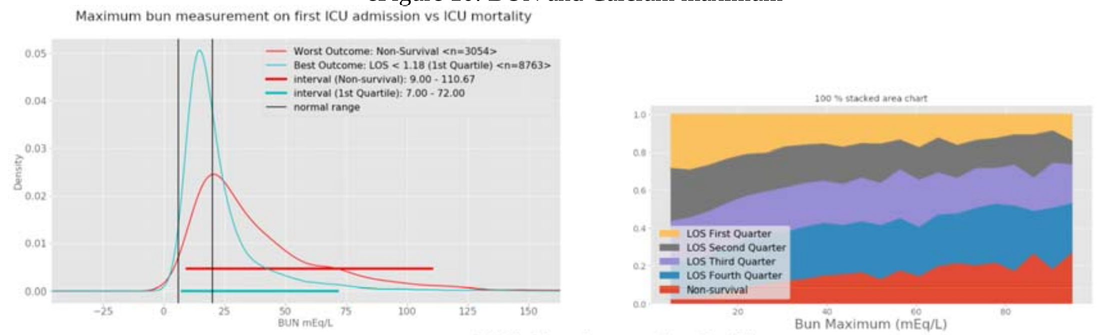

BUN Maximum (mEq/L)

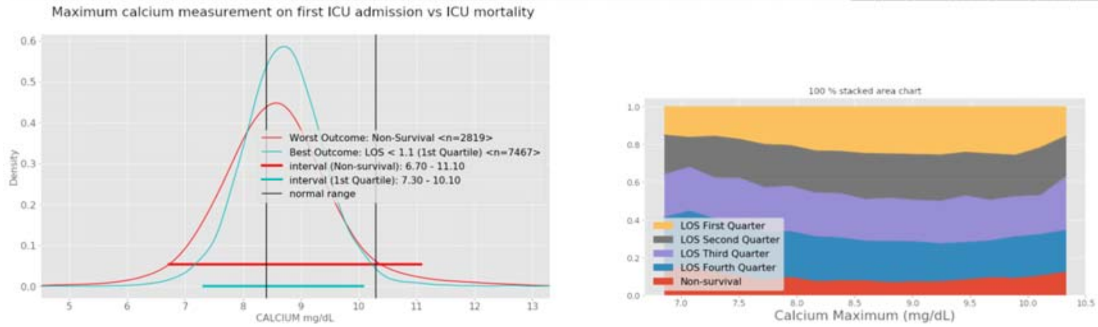

Calcium Maximum (mg/dL)

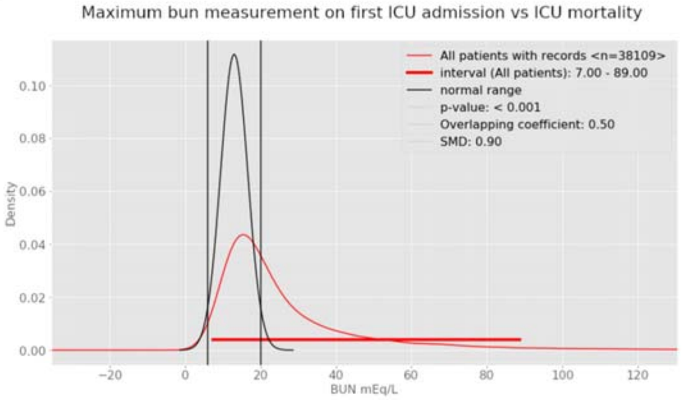

BUN Maximum (mEg/L)

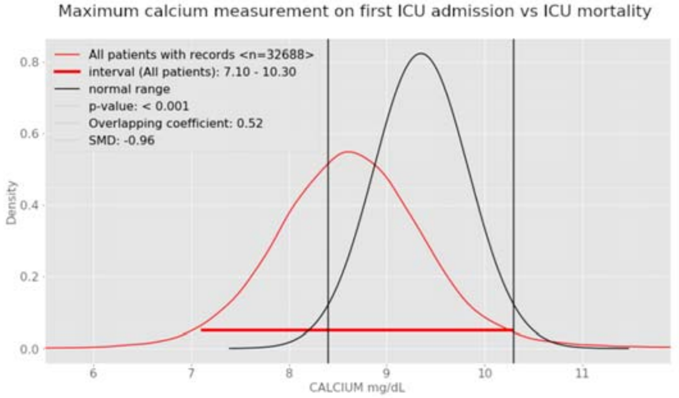

Calcium Maximum (mg/dL)

© 2018 Tyler PD et al. *JAMA Network Open.*

eFigure 11: Chloride and Free Calcium maximum

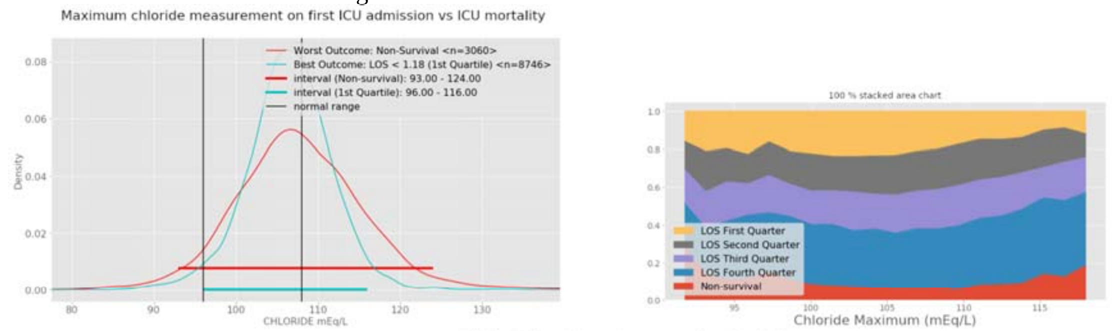

Chloride Maximum (mEq/L)

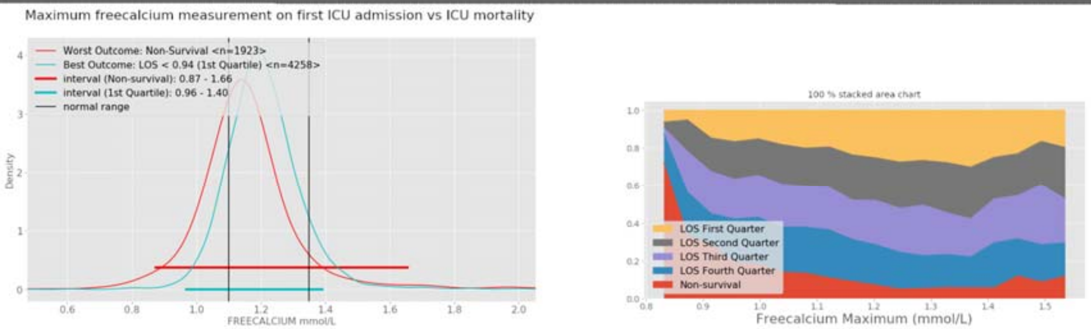

Freecalcium Maximum (mmol/L)

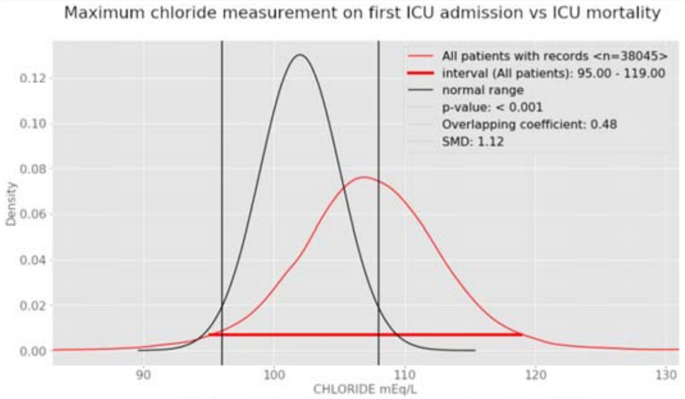

Chloride Maximum (mEg/L)

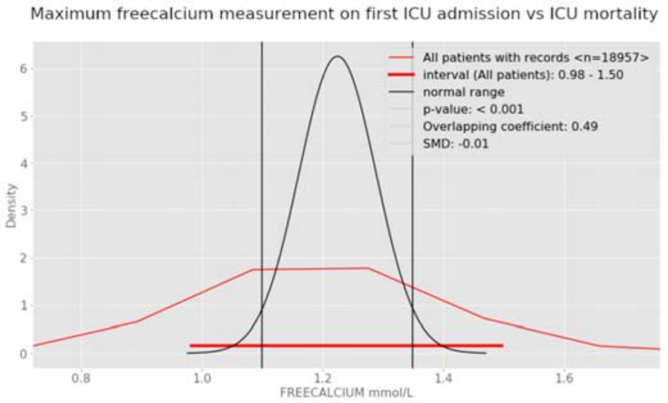

Freecalcium Maximum (mmol/L)

© 2018 Tyler PD et al. *JAMA Network Open.*

eFigure 12: Magnesium and Phosphate maximum

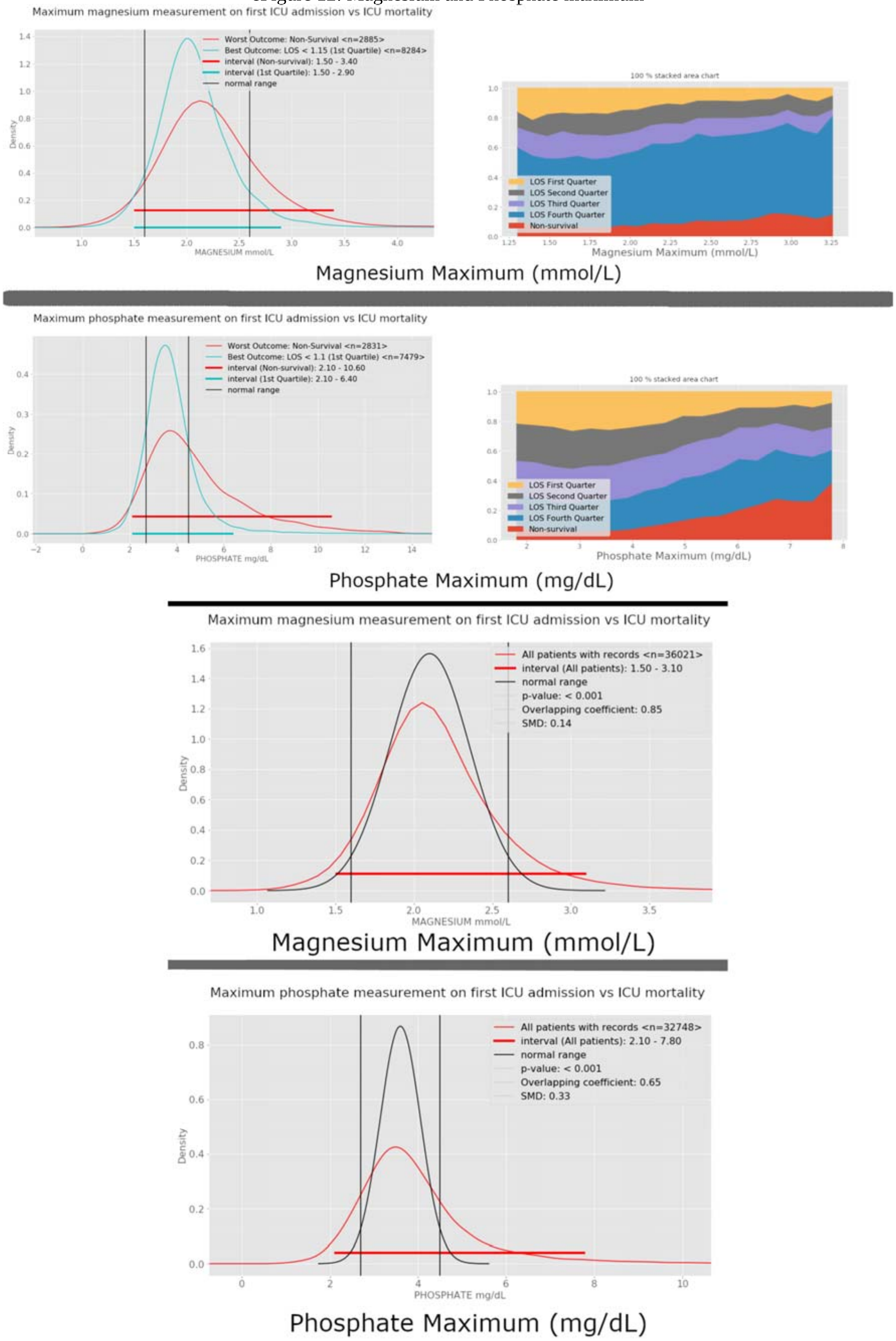

© 2018 Tyler PD et al. *JAMA Network Open.*

eFigure 13: Platelet and Potassium maximum

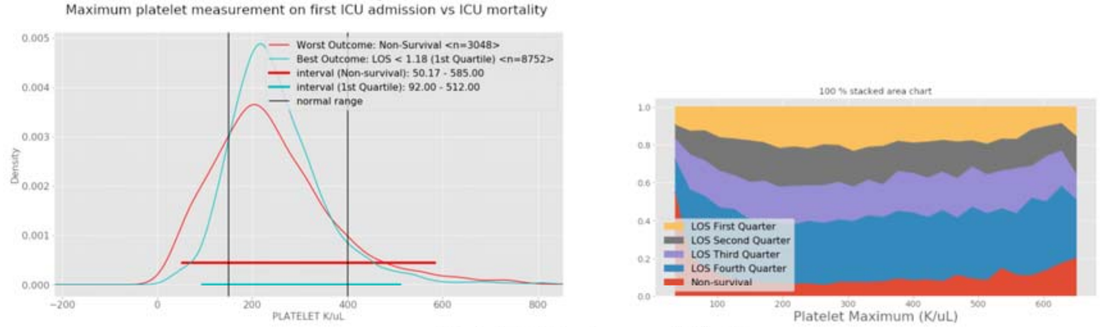

Platelet Maximum (K/uL)

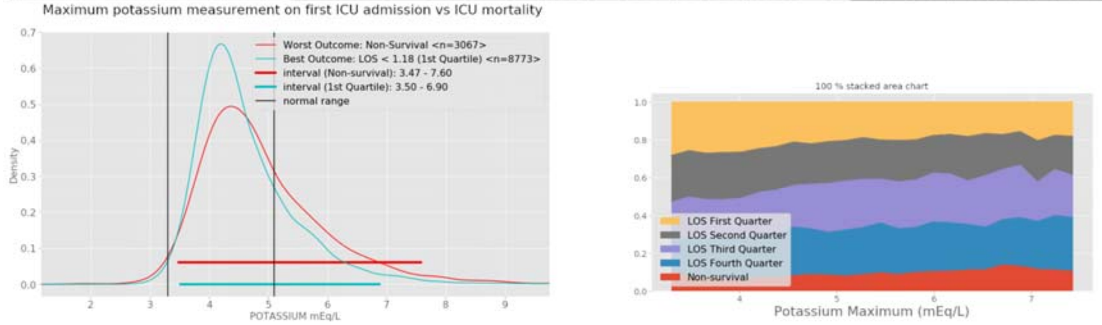

Potassium Maximum (mEq/L)

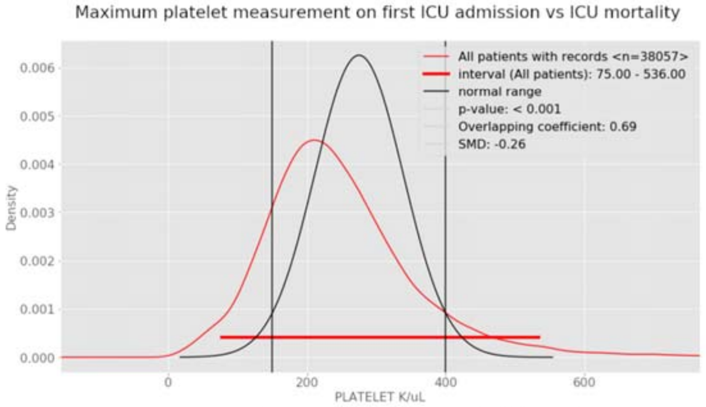

Platelet Maximum (K/uL)

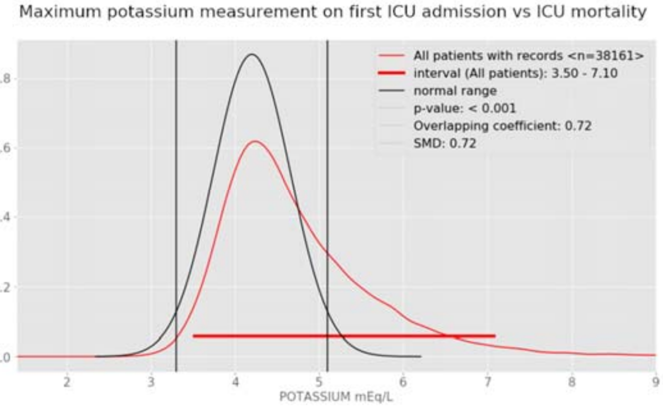

Potassium Maximum (mEq/L)

© 2018 Tyler PD et al. *JAMA Network Open.*

eFigure 14: Sodium and WBC maximum

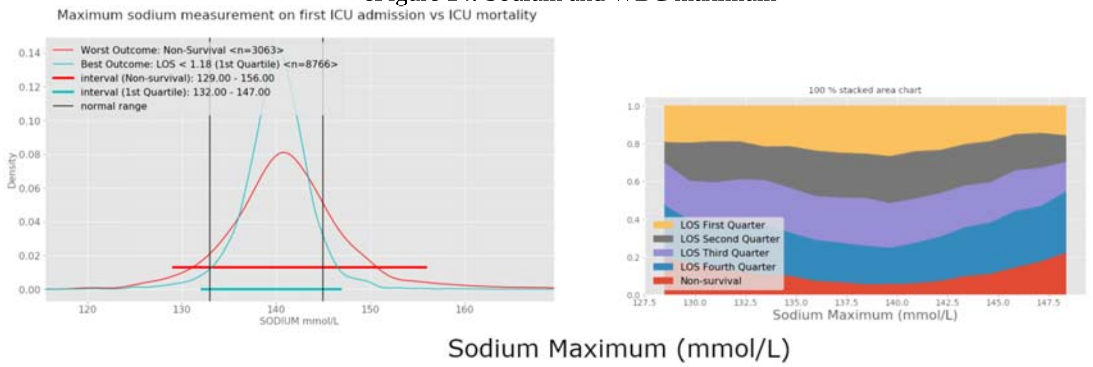

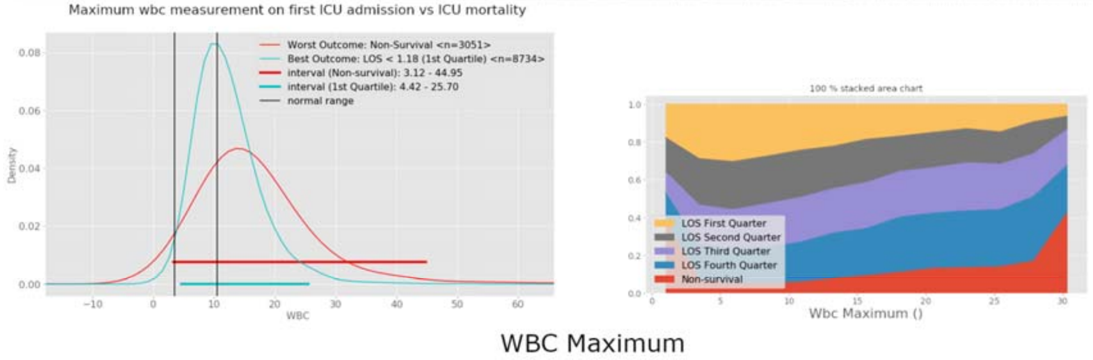

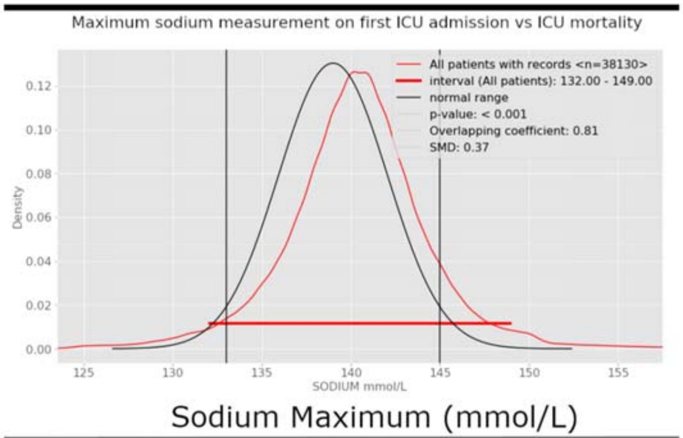

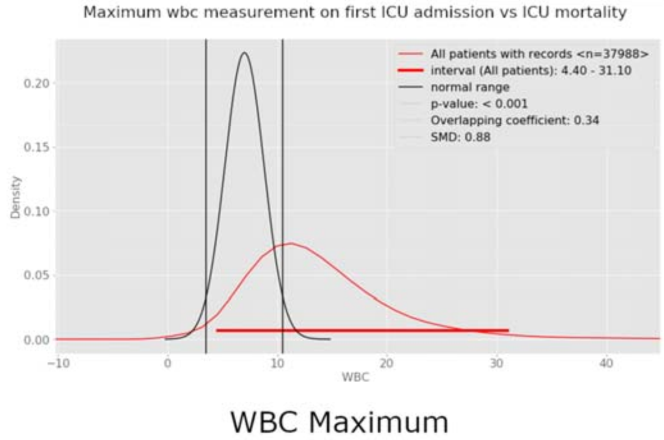

© 2018 Tyler PD et al. *JAMA Network Open.*

eFigure 15: Hemoglobin maximum

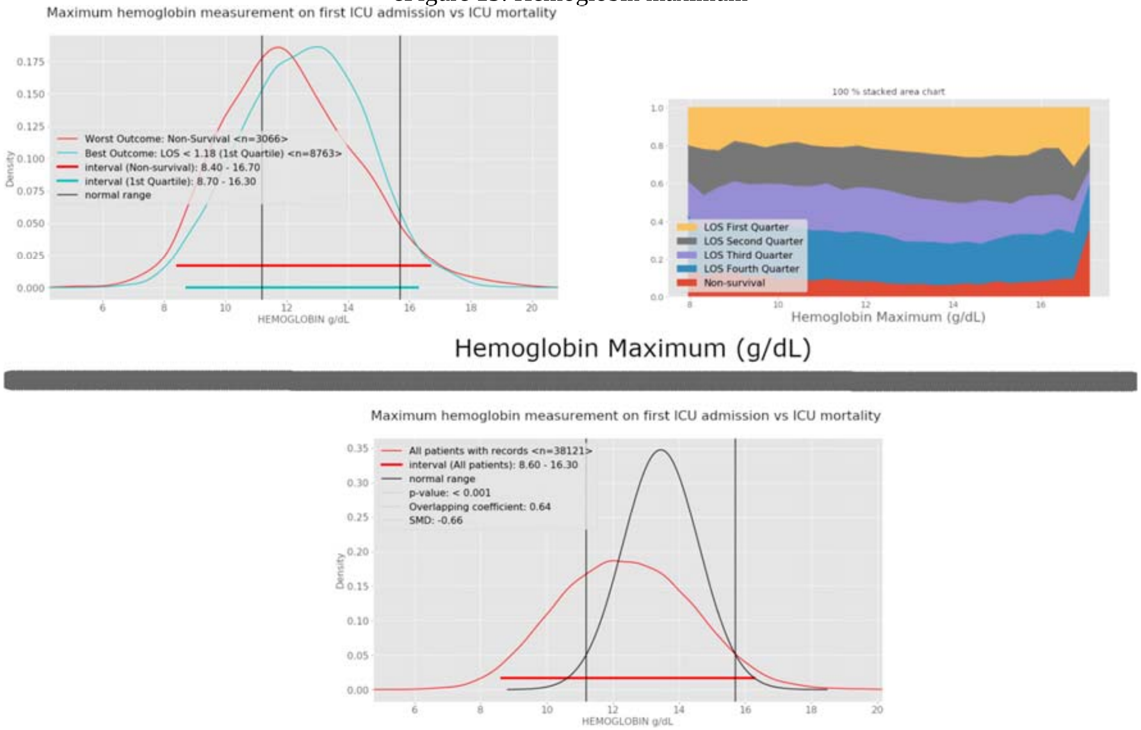

© 2018 Tyler PD et al. *JAMA Network Open.*

Supplementary Tables

More details of Table 2: Overlap Between Laboratory Distributions of all ICU patients, Best Outcome patients, and Worst Outcome patients with the standard reference interval is provided in eTable 1. All numbers are rounded to the third decimal place. Confidence intervals of OVL and SMD are also provided. The confidence intervals were computed using bootstrap method: first, we resampled data from their distribution for 100 times and calculated OVL and SMD respectively. Second step, we ordered OVL and SMD, chose the value at 2.5% percentile as the confidence interval lower bound and the 97.5% as the upper bound. The selected values form 95% confidence interval.

eTable: Overlap Between Laboratory Distributions of all ICU patients, Best Outcome patients, and Worst Outcome patients with the standard reference interval

	All ICU Patients **vs** Standard Reference Interval		Best Outcome Patients **vs** Standard Reference Interval		Worst Outcome Patients **vs** Standard Reference Interval	
	**OVL**	**SMD**	**OVL**	**SMD**	**OVL**	**SMD**
Albumin (Min)	0.311 [0.308, 0.312]	-2.032 [-2.055, -2.026]	0.403 [0.401, 0.413]	-1.686 [-1.707, -1.671]	0.194 [0.189, 0.216]	-2.568 [-2.623, -2.503]
Bicarbonate (Max)	0.723 [0.721, 0.726]	-0.478 [-0.478, -0.467]	0.774 [0.768, 0.777]	-0.405 [-0.418, -0.401]	0.543 [0.533, 0.579]	-0.793 [-0.842, -0.753]
(Min)	0.459 [0.454, 0.463]	-1.253 [-1.261, -1.246	0.531 [0.524, 0.532]	-1.093 [-1.109, -1.089]	0.328 [0.322, 0.356]	-1.583 [-1.640, -1.525]
Bilirubin (Max)	0.496 [0.492, 0.496]	0.898 [0.888, 0.911]	0.574 [0.574, 0.590]	0.763 [0.750, 0.764]	0.284 [0.278, 0.302]	1.284 [1.257, 1.320]
Creatinine (Max)	0.531 [0.527, 0.534]	0.635 [0.626, .650]	0.573 [0.573, 0.605]	0.508 [0.502, 0.517]	0.334 [0.329, 0.357]	1.040 [1.011, 1.043]
Glucose (Max)	0.093 [0.091, 0.095]	1.350 [1.323, 1.385]	0.134 [0.125, 0.134]	1.212 [1.205, 1.300]	0.077 [0.076, 0.080]	1.607 [1.568, 1.687]
(Min)	0.399 [0.395, 0.402]	0.938 [0.935, 0.956]	0.417 [0.414, 0.419]	0.960 [0.955, 0.966]	0.287 [0.285, 0.301]	0.932 [0.913, 0.965]
Hemoglobin (Min)	0.326 [0.326, 0.329]	-1.802 [-1.813, -1.794]	0.398 [0.398, 0.403]	-1.538 [-1.540, -1.534]	0.302 [0.297, 0.327]	-1.812 [-1.882, -1.743]
Lactate (Max)	0.406 [0.403, 0.406]	0.989 [0.986, 0.989]	0.467 [0.463, 0.471]	0.951 [0.912, 0.957]	0.234 [0.232, 0.241]	1.278 [1.271, 1.307]
Magnesium (Max)	0.854 [0.852, 0.860]	0.135 [0.122, 0.143]	0.857 [0.856, 0.861]	-0.040 [-0.051, -0.031]	0.734 [0.718, 0.756]	0.236 [0.197, 0.247]
(Min)	0.611 [0.606, 0.614]	-0.839 [-0.845, -0.826]	0.650 [0.647, 0.654]	-0.807 [-0.818, -0.799]	0.591 [0.591, 0.610]	-0.719 [-0.746, -0.704]
Phosphate (Max)	0.650 [0.650, 0.655]	0.331 [0.323, 0.341]	0.705 [0.705, 0.716]	0.163 [0.161, 0.173]	0.461 [0.461, 0.467]	0.790 [0.790, 0.824]
(Min)	0.574 [0.572, 0.576]	-0.423 [-0.437, 0.412]	0.632 [0.632, 0.642]	-0.428 [-0.434, -0.405]	0.485 [0.485, 0.523]	0.035 [0.028, 0.083]

© 2018 Tyler PD et al. *JAMA Network Open.*

Platelet count (Min)	0.525 [0.522, 0.531]	-0.873 [-0.876, -0.857]	0.575 [0.571, 0.577]	-0.753 [-0.773, 0.750]	0.481 [0.473, 0.507]	-0.916 [-0.950, -0.834]
Potassium (Max)	0.722 [0.719, 0.725]	0.723 [0.722, 0.729]	0.774 [0.769, 0.775]	0.611 [0.602, 0.628]	0.649 [0.639, 0.669]	0.823 [0.774, 0.851]
(Min)	0.584 [0.580, 0.588]	-0.991 [-1.004, -0.969]	0.618 [0.610, 0.620]	-0.907 [-0.915, -0.897]	0.622 [0.611, 0.647]	-0.706 [-0.764, -0.653]
Sodium (Max)	0.806 [0.806, 0.812]	0.368 [0.360, 0.375]	0.826 [0.818, 0.837]	0.314 [0.309, 0.341]	0.687 [0.679, 0.706]	0.451 [0.441, 0.505]
(Min)	0.716 [0.713, 0.715]	-0.656 [-0.668, -0.661]	0.767 [0.759, 0.773]	-0.594 [-0.604, -0.579]	0.681 [0.681, 0.710]	-0.587 [-0.605, -0.535]
WBC Count (Max)	0.340 [0.338, 0.346]	0.881 [0.827, 1.002]	0.411 [0.410, 0.415]	0.776 [0.738, 0.870]	0.256 [0.256, 0.266]	0.694 [0.587, 0.741]
(Min)	0.586 [0.584, 0.589]	0.569 [0.524, 0.659]	0.636 [0.643, 0.648]	0.507 [0.483, 0.545]	0.396 [0.380, 0.410]	0.499 [0.436, 0.602]
Calcium (Max)	0.520 [0.514, 0.524]	-0.955 [-0.983, -0.924]	0.552 [0.552, 0.559]	-1.019 [-1.031, -0.996]	0.488 [0.488, 0.516]	-0.690 [-0.790, -0.690]
(Min)	0.333 [0.333, 0.338]	-1.704 [-1.711, -1.696]	0.391 [0.389, 0.397]	-1.558 [-1.567, -1.541]	0.274 [0.270, 0.300]	-1.758 [-1.849, -1.717]
Ionized Calcium (Min)	0.258 [0.258, 0.267]	-1.833 [-1.843, -1.823]	0.266 [0.266, 0.276]	-1.966 [-1.979, -1.939]	0.262 [0.255, 0.286]	-1.655 [-1.724, -1.622]

© 2018 Tyler PD et al. *JAMA Network Open.*
